# Whole-Genome Sequencing of *Sordaria macrospora* Mutants Identifies Developmental Genes

**DOI:** 10.1534/g3.111.001479

**Published:** 2012-02-01

**Authors:** Minou Nowrousian, Ines Teichert, Sandra Masloff, Ulrich Kück

**Affiliations:** Lehrstuhl für Allgemeine und Molekulare Botanik, Ruhr-Universität Bochum, 44780 Bochum, Germany

**Keywords:** next-generation sequencing, developmental mutants, *Sordaria macrospora*

## Abstract

The study of mutants to elucidate gene functions has a long and successful history; however, to discover causative mutations in mutants that were generated by random mutagenesis often takes years of laboratory work and requires previously generated genetic and/or physical markers, or resources like DNA libraries for complementation. Here, we present an alternative method to identify defective genes in developmental mutants of the filamentous fungus *Sordaria macrospora* through Illumina/Solexa whole-genome sequencing. We sequenced pooled DNA from progeny of crosses of three mutants and the wild type and were able to pinpoint the causative mutations in the mutant strains through bioinformatics analysis. One mutant is a spore color mutant, and the mutated gene encodes a melanin biosynthesis enzyme. The causative mutation is a G to A change in the first base of an intron, leading to a splice defect. The second mutant carries an allelic mutation in the *pro41* gene encoding a protein essential for sexual development. In the mutant, we detected a complex pattern of deletion/rearrangements at the *pro41* locus. In the third mutant, a point mutation in the stop codon of a transcription factor-encoding gene leads to the production of immature fruiting bodies. For all mutants, transformation with a wild type-copy of the affected gene restored the wild-type phenotype. Our data demonstrate that whole-genome sequencing of mutant strains is a rapid method to identify developmental genes in an organism that can be genetically crossed and where a reference genome sequence is available, even without prior mapping information.

The analysis of mutants with a distinct phenotype that were generated, for example, by random mutagenesis, is a powerful approach to identify factors essential for biological processes. This so-called forward genetics approach is important both in basic research as well as for medical applications, *e.g.* for the identification of mutations that underlie diseases. However, identifying the mutation that causes a phenotype is often time-consuming and requires additional resources, *e.g.* previous knowledge about genetic/physical markers for genetic linkage analysis or genomic/cDNA libraries for complementation experiments. In recent years, so-called next-generation sequencing techniques were developed that allow a massively increased sequence throughput at greatly reduced costs (Metzker 2010; Nowrousian 2010; Shendure and Li 2008), which now enable a different strategy, namely the sequencing of mutant genomes to identify affected genes. Strategies for mutation identification through whole-genome sequencing have been used for several model organisms, including *Schizosaccharomyces pombe*, *Caenorhabditis elegans*, *Arabidopsis thaliana*, *Neurospora crassa*, the rodent malaria parasite *Plasmodium chabaudi*, and humans (Flibotte *et al.* 2010; Irvine *et al.* 2009; Lupski *et al.* 2010; McCluskey *et al.* 2011; Pomraning *et al.* 2011; Puente *et al.* 2011; Roach *et al.* 2010; Sarin *et al.* 2008; Schneeberger *et al.* 2009; Zuryn *et al.* 2010). This indicates that this approach is feasible even for large eukaryotic genomes; however, the strategies used in these studies often included previous mapping of the mutation to a small genomic region through the use of single nucleotide polymorphism (SNP) markers, crosses of the mutant strain to a strain with a different genetic background, or resequencing of large numbers of candidate mutations to distinguish between true mutations and errors in the sequenced or the reference genomes. Therefore, the studies have been largely restricted to organisms in which genetic and/or genomic resources that assist in mapping are already available.

The aim of our study was to determine whether whole-genome sequencing of mutant strains is a useful approach to identify developmental genes in a filamentous fungus for which such resources are not available. Our experimental system is *Sordaria macrospora*, a model organism for the analysis of fungal multicellular development and meiosis (Engh *et al.* 2010; Kück *et al.* 2009; Zickler 2005). A set of genetic and molecular tools has been established for this fungus (Nowrousian *et al.* 2005; Pöggeler and Kück 2006; Pöggeler *et al.* 1997, 2003; Walz and Kück 1995), and more than 100 developmental mutants were generated through ultraviolet or ethyl methanesulfonate (EMS) mutagenesis (Kück *et al.* 2009), seven of which have already been characterized using classical complementation analysis, leading to the identification of novel developmental genes (Bloemendal *et al.* 2010; Engh *et al.* 2007b; Kück 2005; Masloff *et al.* 1999; Nowrousian *et al.* 1999, 2007; Pöggeler and Kück 2004). Recently, the *S. macrospora* genome was sequenced and assembled from next-generation sequence reads (Nowrousian *et al.* 2010); however, SNP maps, other large-scale physical or genetic mapping data, or characterized strains with different genetic background are not available for *S. macrospora*.

In this study, we describe the identification of the causative mutations in three developmental mutants via a strategy involving whole-genome sequencing of pooled DNA from 40 single spore isolates per strain of three mutants and the wild type, in addition to subsequent bioinformatics analyses. The data presented here demonstrate that this strategy allows rapid identification of the developmental genes affected in the mutant strains, is feasible for an organism that can be genetically crossed and for which a reference genome is available, and does not require prior mapping information.

## Materials and Methods

### Strains, culture conditions, mutagenesis, and genetic crosses

*S. macrospora* strains (Table 1) were grown on corn meal agar as described previously (Esser 1982). Generation of developmental mutants by EMS mutagenesis was as described by Pöggeler and Kück (2004). For crosses, strains were inoculated on opposite sides of a Petri dish, and asci were collected from perithecia formed in the contact zone of the two mycelia. A complete crossing history of the mutants used in this study is presented in supporting information, Figure S1. For transcript analyses, strains were grown in minimal medium as described (Nowrousian *et al.* 2005).

**Table 1:** *S. macrospora* strains used in this study

Strain	Phenotype	Genotype	Reference
Wild type k-hell	Wild type	Wild type	FGSC 10222
pro23 S43911	Sterile	pro23	DGMB*^a^*
pro44 S94061	Sterile	pro44	DGMB*^a^*
fus1-1 S84595	Light-brown ascospores	fus	DGMB*^a^*
r2 S67813	Brown-red ascospores	r2	DGMB*^a^*

a Culture collection of the Department of General and Molecular Botany.

### DNA preparation and Illumina/Solexa sequencing

For the preparation of pooled DNA from 40 single spore isolates, all strains were grown separately, then equal amounts of mycelium (equal weight after removal of medium by filtering) were combined, frozen in liquid nitrogen, and pulverized for subsequent DNA extraction as described previously (Nowrousian *et al.* 2010). Genomic DNA (5 µg) was used for Illumina/Solexa sequencing with a GAII or HiSeq at GATC Biotech (Konstanz, Germany). Reads of 50 or 76 bases were obtained from both ends of 2-kb fragments using a mate-pair sequencing strategy (Korbel *et al.* 2007). For DNA from spores representing the wild type and pro23/fus, respectively, one GAII lane of mate-pair reads was obtained for each sample. For DNA from spores representing fus, one GAII lane of single reads and two lanes of mate-pair reads were obtained, and for DNA from pro44, one HiSeq lane of mate-pair reads was obtained (for overview of read numbers, see Table 2).

**Table 2:** Summary of sequence reads generated from mutant and wild-type samples

Sample	Total No. of Reads	Read Length in Bases	Total Mb	Coverage	No. of Reads Mapped to Reference Genome	% of Reads Mapped to Reference Genome
Wild type	28,760,231	76	2186	54x	17,322,318	60.2
pro23/fus	23,164,874	76	1761	44x	16,616,121	71.7
fus	56,139,346	76	4267	107x	48,548,370	86.5
pro44	86,849,208	50	4342	109x	73,788,312	85.0

The total number of reads is the number after removing reads containing undefined bases (“N”). Reads were mapped to the *S. macrospora* reference genome (Nowrousian *et al.* 2010) using the Burrows-Wheeler Alignment tool (BWA) (Li and Durbin 2009).

### Cleaning of raw sequence reads, mapping of reads to the reference genome, and analysis of sequence variants

Sequence reads that contained undetermined bases (“N”) were removed using custom-made Perl scripts. Reads lacking a proper mate after the cleaning process were collected in separate files and treated as single reads in the subsequent mapping. The cleaned reads were mapped to the *S. macrospora* reference genome (Nowrousian *et al.* 2010) using the Burrows-Wheeler Alignment tool (Li and Durbin 2009). The resulting SAM files were processed using the SAMtools pileup and filtering functions (Li *et al.* 2009). For a detailed description, see File S1. To identify putative mutations that were present in one or two strains only, pileup results were processed with custom-made Perl scripts. To analyze regions present in the reference genome but not covered in the sequenced mutant strains (putative deletions), a consensus sequence for the sequenced strains was extracted with pileup (Li *et al.* 2009), and subsequently screened for regions not covered in the mutant strains using custom-made Perl scripts. Another approach to test for putative insertions, deletions, or inversions is to search for deviations from the expected distance of paired reads. This was performed using the SAM files from BWA and custom-made Perl scripts (File S1).

### RNA preparation and expression analysis

Strains were grown under conditions that promote sexual development. RNA was prepared and reverse transcription was performed as described previously (Nowrousian *et al.* 2005).

### Cloning of plasmids for complementation of mutant strains

Plasmids for complementation of mutant pro44 were generated by standard cloning techniques, or by homologous recombination in yeast as described by Colot *et al.* (2006). Plasmid pIG3146-37 contains the *pro44* (*SMAC_03223*) open reading frame (ORF; including introns), and 1.1 kb of upstream as well as 0.8 kb of downstream genomic regions in pDrive (QIAGEN, Hilden, Germany). Plasmid pIG3147-1 contains the *pro44* ORF under control of the *A. nidulans gpd* promoter and *trpC* terminator in vector pEHN8, which contains a hygromycin B resistance cassette for selection in *S. macrospora* (I. Teichert and U. Kück, unpublished data). Plasmid pRSnat-pro44-NA contains the *pro44* (*SMAC_03223*) ORF (including introns), and 1.5 kb of upstream as well as 1.5 kb of downstream genomic regions in pRSnat, which contains a nourseothricin resistance cassette for selection in *S. macrospora* (Klix *et al.* 2010). Plasmid pRSnat-pro44-OE contains the *pro44* ORF under control of the *A. nidulans gpd* promoter and *trpC* terminator in vector pRSnat. Plasmid pEHN5-tih for the fus mutant complementation contains the *tih* ORF under control of the *A. nidulans gpd* promoter and *trpC* terminator in plasmid pEHN5 carrying *hph* as a selectable marker (Teichert and Kück, unpublished).

### Transformation of *S. macrospora*

*S. macrospora* was transformed as described (Nowrousian *et al.* 1999) with the following modifications: Protoplasts were generated from vegetative mycelium using 40 mg/ml Glucanex 100 G (Novozymes A/S, Bagsvaerd, Denmark) and 1.4 U/ml chitinase (ASA Spezialenzyme GmbH); transformants were selected on 80 U/ml hygromycin B or 50 U/ml nourseothricin. Plasmid pIG3146-37 does not contain a resistance marker for selection in *S. macrospora* and was therefore co-transformed with plasmid pEHN1 that contains a Hygromycin B resistance cassette (Teichert and Kück, unpublished).

### Accession numbers

The sequence reads that were generated in this study were submitted to the NCBI sequence read archive (accession number SRA026818.1). The updated *S. macrospora* genome assembly (version 02) was submitted to the EMBL database (ENA) and is available under accession numbers CABT02000001-CABT02001583 and at http://c4-1-8.serverhosting.rub.de/public/.

## Results

### Sequencing of pooled genomic DNA and mapping to the reference genome

The pro23, pro44, and fus mutant strains used in this study were generated in a previous mutagenesis program (Kück *et al.* 2009; Masloff *et al.* 1999). The mutants pro23 and pro44 are sterile; they form only fruiting body precursors (protoperithecia) but no mature perithecia or ascospores. Mutant fus (*fusca*, Latin for brown) is fertile but generates light-brown instead of black ascospores and secretes a red-brown pigment into the medium. During the initial mutant screen, mutant phenotypes were tested for Mendelian segregation via backcrosses against the wild type, and single spore isolates were used for further analyses. When we initiated this study, each mutant had been backcrossed several times (Figure S1), and the successive rounds of crosses should already have reduced the mutational load acquired during mutagenesis.

**Figure 1:**
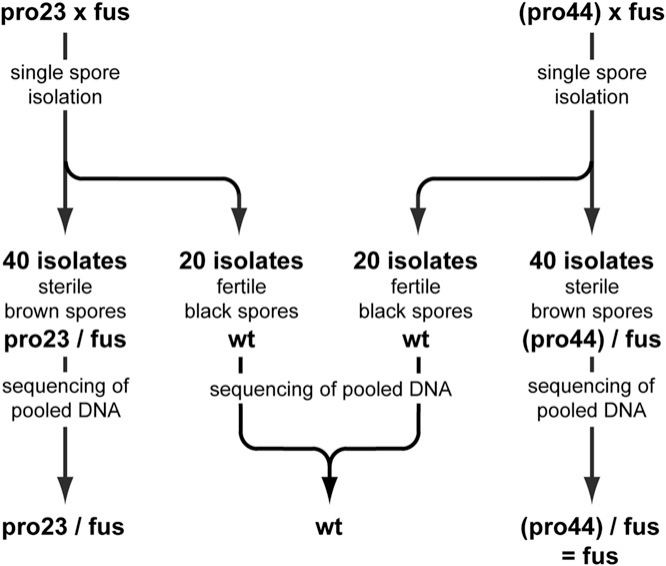
Strategy for whole-genome sequencing of pooled DNA from mutants and wild type. Mutants pro23 and pro44 were crossed against the spore color mutant fus. Single spore isolates derived from black and light-brown ascospores were screened for fertility and color; for both crosses, 40 spore isolates with a sterile/light-brown phenotype were chosen to represent double mutants pro23/fus and pro44/fus, respectively. For the resequencing of the wild type, 20 isolates with a fertile/black phenotype were chosen from each cross. The pooled DNA from 40 spore isolates for each genotype was used for sequencing. As it turned out during the analysis, mutant pro44 had acquired a second, unrelated mutation that also led to sterility. Therefore, the mutant name is given in brackets to indicate that this sample was no longer used for the analysis of pro44 but only for the analysis of the fus mutation. For the pro44 analysis, the mutant was outcrossed again to obtain a clean pro44 strain; this strain was used for crossing to obtain single spore isolates for sequencing according to the same principle (Figure S2).

However, to ensure that the genomic DNA for whole-genome sequencing was relatively homogenous, and that we would be able to distinguish between true mutations and sequencing errors, we used the following strategy (Figure 1): Mutants pro23 and pro44 were crossed against the fus mutant (sterile mutants of the homothallic *S. macrospora* are usually fertile when outcrossed against a strain carrying the wild type-allele of the affected gene). Recombinant asci from these crosses can be readily distinguished because they contain four black and four light-brown ascospores. From each cross, 40 single spore isolates with the phenotype “light-brown spores, sterile” corresponding to the respective genotypes pro23/fus and pro44/fus were isolated. In addition, 20 single spores exhibiting a “black spores, fertile” phenotype corresponding to the wild-type genotype were isolated from each cross. Pooled genomic DNA from the 40 spore isolates from each of the three genotypes was used for Illumina/Solexa sequencing.

As it turned out during the analysis, mutant pro44 had spontaneously acquired a second, unrelated mutation also resulting in sterility. Spontaneous mutations occur with varying frequencies in fungi (Drake 1999). Therefore, the pro44/fus sample was analyzed only with respect to the fus genotype, and is from this point denoted as “fus.” For the pro44 mutation analysis, pro44 was outcrossed again to obtain a clean pro44 strain, and this strain was used in a subsequent cross to obtain single spore isolates for sequencing, according to the same principle as described above (Figure S2). Consequently, the sequenced DNA pools correspond to the four samples wild type, pro23/fus, fus, and pro44 (Table 2).

For each of the four genotypes, one or two lanes of mate-pair reads were obtained, corresponding to a 40- to 100-fold coverage of the 40 Mb *S. macrospora* genome (Table 2). Mate-pair sequencing was used to facilitate detection of larger insertion/deletion events (indels) by analyzing the distance between mapped reads of a pair (Korbel *et al.* 2007). We chose this sequencing strategy because our previous analyses of strains from EMS mutagenesis showed that the causative mutations range from point mutations (Bloemendal *et al.* 2010; Engh *et al.* 2007b; Pöggeler and Kück 2004) to deletions of several kilobases (Nowrousian *et al.* 2007). Before mapping, reads with ambiguous bases were removed, which left 23 to 86 million usable reads per sample (Table 2). Reads were mapped to the *S. macrospora* reference genome using BWA (Li and Durbin 2009) (File S1). From each sample, 60% to 87% of the reads mapped to the genome (Table 2), consistent with results in other systems, *e.g.* 71% in a *C. elegans* mutant sequencing project (Sarin *et al.* 2008).

### Identification of mutations in the sequenced strains through bioinformatics analyses

The four sequenced DNA samples correspond to the pro23/fus, fus, pro44, and wild-type genotypes. Therefore, the causative mutation for the pro23 phenotype should occur with 100% penetrance, *i.e.* present in all reads covering the corresponding position(s), in the pro23/fus sample only, but not in the pro44, fus, or wild-type samples. The same is true for the causative mutation in pro44, whereas the mutation causing the fus phenotype should be present with 100% penetrance in the pro23/fus and fus samples, but not in the wild-type or pro44 sample. Deviations from the reference sequence that are present in only a few reads of one or more strains are most likely caused by sequencing errors or represent mutations that are present in the genomes of some of the single spore isolates used for sequencing but are not the causative mutations for pro23, pro44, or fus. Therefore, we analyzed the mapping results for deviations from the reference sequence in only one sample, in combinations of two samples, or in all samples (Table 3, File S1).

**Table 3:** Summary of small sequence variants detected for the sequenced genotypes when compared with the reference genome

Genotype	Sequenced Sample(s)	Number of Small Variants With Coverage > 40%	No. of These With 100% Penetrance	Location of Putative Mutation
Wild type	wild type	97	0	–
pro23	pro23/fus	139	0	–
pro44	pro44	386	7	6 variants not within coding region, one changes stop codon of *SMAC_03223*
fus	pro23/fus and fus	545	1	changes intron splice site of *tih*

Mutations caused by small sequence variants were identified by screening the sequence data for SNPs and indels (insertions/deletions) of <4 bases with a coverage of at least 40% of the average coverage (see Table 2) for that sample, and which occur in one sample only, or in the case of the fus mutant, in two samples (pro23/fus and fus). For these putative mutations, it was subsequently checked whether they had 100% penetrance, *i.e.* all the reads in the strain had the SNP/indel and none of the reads in another strain carried this specific mutation.

In principle, mutations can be caused by a number of genomic differences, *e.g.* SNPs and small indels of a few bases, or structural variants. The latter include large indels, inversions, more complex rearrangements involving combinations of insertions, deletions and inversions, and translocations. Different forms of aneuploidy can also cause mutant phenotypes, but many forms of aneuploidy as well as translocations of chromosome arms result in problems during meiosis. Therefore, it seemed unlikely that these types of mutations would be the underlying mutations here because all of our mutants form normal asci when outcrossed indicating meiosis is unaffected (data not shown). Thus, we looked for the following types of variants: SNPs and small indels of a few bases, and larger insertions or deletions in the range of several kilobases.

To identify mutations caused by small sequence variants, sequence data were screened for SNPs and indels of <4 bases that had a read coverage of at least 40% of the average coverage for the specific strain, and occurred in one sample only, or in the case of the fus mutant, in two samples (pro23/fus and fus; Table 3). The coverage threshold was applied because random verification of several putative mutations as well as coverage and variant frequency analysis (Figure S3) indicated that regions with low sequence coverage did not allow for reliable detection of variants; these regions are probably difficult to sequence, *e.g.* because of repetitive sequences, or the formation of secondary DNA structures. A similar strategy was used successfully in a *C. elegans* mutant sequencing project (Flibotte *et al.* 2010). The resulting putative sequence variants were screened for 100% penetrance, *i.e.* all the reads in the sample contained the SNP/indel, and none of the reads in a nonrelated sample carried this specific mutation. In the case of the fus mutant, exactly one mutation was found that satisfied these conditions, and this turned out to be the causative mutation (Table 3; see section *Analysis of the causative mutation in mutant fus*). For the pro44 mutant, seven mutations were detected that satisfied these conditions, and only one of these changed the coding region of a gene and was established as the causative mutation (see section *Analysis of the causative mutation in mutant pro44*).

The low number of reliably detected SNPs and small indels was not the result of insufficient coverage or lack of sequencing quality because we detected 1193 variants present in all sequenced samples that presumably represent errors in the reference sequence. Most of these variants were single-base indels within sequence regions consisting of repeats of one base, and therefore they most likely represent homopolymer errors introduced through 454 sequence reads (Balzer *et al.* 2011) that were used for the assembly of the reference genome (Nowrousian *et al.* 2010). Polymerase chain reaction (PCR) amplification and sequencing of several cases confirmed the sequence error in the reference genome (data not shown). The 1193 errors were corrected; and in addition, the wild type mate-pair information was used to connect contigs, reducing the contig number in the *S. macrospora* genome from 4781 to 1583. The improved genome assembly was released as genome version 02 (acc. no. CABT02000001-CABT02001583, http://c4-1-8.serverhosting.rub.de/public/).

Because our small variant analysis did not detect the causative mutation for pro23, we searched for larger indels in the sequenced samples. We used the following two strategies for this purpose: (1) we identified regions that were not covered by sequence reads in the pro23/fus sample but were covered in the other samples; and (2) we identified genomic regions in which the predicted insert size between the two reads of a mate-pair from the mapping data deviated significantly from the expected insert size of the sequenced library (File S1).

The coverage analysis resulted in the detection of a 1.1-kb region not covered in the pro23/fus sample, but well-covered in the other samples. This deleted region contained part of the open reading frame (ORF) of the developmental gene *pro41*, and the variant was established as the causative mutation of mutant pro23 (as mentioned the section *Analysis of the causative mutation in mutant pro23*). Interestingly, analysis of mate-pair insert sizes did not detect the deletion. The reason for this turned out to be that this region in mutant pro23 apparently underwent complex rearrangements that resulted in the substitution of a 1.1-kb region that included part of *pro41* by another genomic region of similar size, but unrelated sequence (see below). Thus, searching for noncovered regions and analyzing mate-pair information does not necessarily give the same results; instead, the two analyses can be complementary. Overall, we were able to identify two point mutations and one insertion/deletion event as the causative mutations for the three mutants.

### Analysis of the causative mutation in mutant pro23

**Figure 2:**
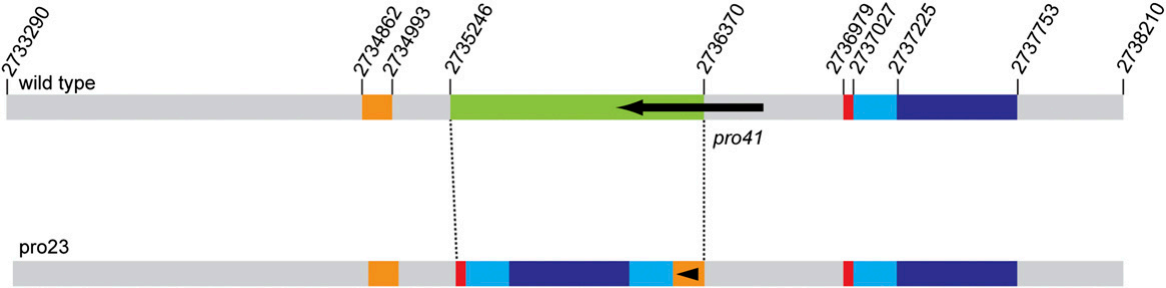
Genomic structure of the *pro41* gene locus in the wild-type and mutant pro23. Numbers in the wild type correspond to nucleotides in contig 2.1 (*S. macrospora* genome version 02). An approximately 1.1-kb region (green in the wild type) is deleted in mutant pro23. Several other regions (orange, red, and light and dark blue) adjacent to the deleted region are duplicated in the mutant strain and occupy the position of the deleted region. In most cases, the orientation of the duplicated regions is the same as in the wild type, with the exception of one case in which a small region is inverted in pro23 (indicated by an arrowhead within the duplicated region). The deleted region comprises the C-terminal part of *pro41* (black arrow).

The analysis of genomic regions not covered by sequence reads from sample pro23/fus, but covered by reads in the other samples yielded a 1.1-kb genomic region that contained part of the *pro41* ORF. Southern blot analysis using part of the region that is deleted in mutant pro23 as a probe showed that indeed this region is present in the wild-type and mutant pro44, but absent in mutant pro23 (data not shown). The region around the *pro41* gene was PCR-amplified from genomic DNA of mutant pro23 and resequenced to confirm the genomic structure in the mutant strain. Interestingly, the genomic region that contains *pro41* is not only partly deleted in mutant pro23 but carries a complex set of rearrangements involving duplications and inversions of several adjacent regions that occupy the space of the deleted 1.1-kb fragment (Figure 2).

**Figure 3:**
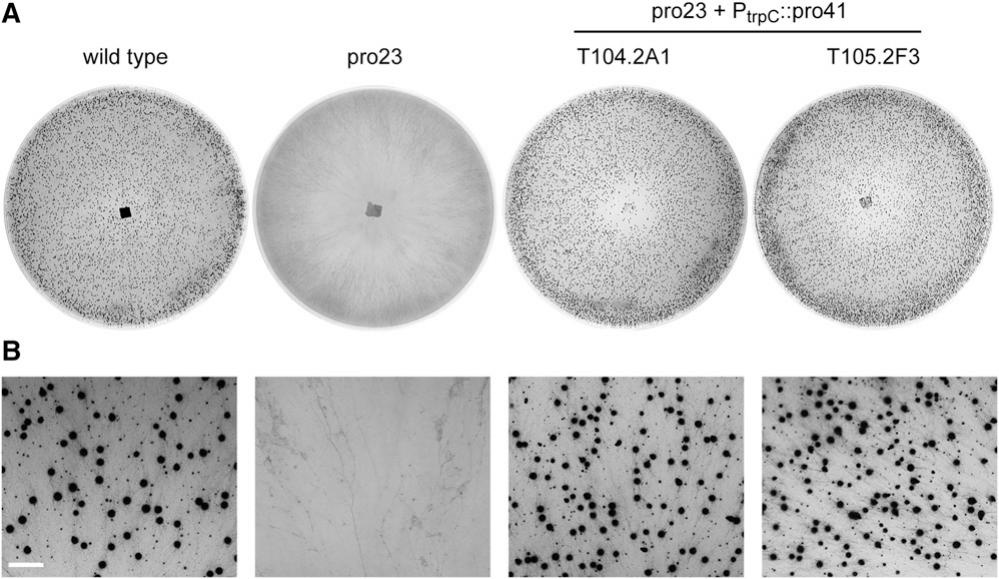
Complementation of mutant pro23 with the *pro41* ORF. Mutant pro23 was transformed with plasmid pE3-5Mr that carries the *pro41* ORF under control of the respective *gpd* and *trpC* promoter and terminator sequences of *A. nidulans*. The figure shows the wild-type (forms perithecia; black dots in the photographs of the petri dishes), mutant pro23 (sterile, does not form perithecia), and two complemented transformants (produce perithecia). Strains were grown on corn meal agar, and photographs were taken after 6 d (A) or 8 d (B). Scale bar in (B) indicates 2 mm.

*pro41* encodes an endoplasmic reticulum membrane protein that is essential for sexual development of *S. macrospora* (Nowrousian *et al.* 2007). The deleted region in mutant pro23 contains part of the *pro41* ORF (Figure 2), and it was shown previously that this part is essential to complement mutant pro41, which carries a deletion comprising the complete *pro41* ORF (Nowrousian *et al.* 2007). Therefore, it seemed likely that the absence of this part of the *pro41* gene causes sterility in mutant pro23. To verify this hypothesis, the mutant was transformed with a construct containing the *pro41* ORF, previously shown to complement mutant pro41 (Nowrousian *et al.* 2007). The resulting transformants were fertile, producing mature fruiting bodies (perithecia) similar to the wild type (Figure 3). These results demonstrate that the lack of a functional *pro41* is the causative mutation in mutant pro23.

### Analysis of the causative mutation in mutant fus

Bioinformatics analysis of small variants yielded a single point mutation (G to A transition) that was present in pro23/fus and fus but not in the wild type and pro44 samples. Analysis of annotated features at the position of the mutation showed that the mutation alters the first nucleotide of the second intron of the *tih* gene encoding trihydroxynaphtalene reductase (locus tag *SMAC_05650*). TIH was predicted to be part of the melanin biosynthesis pathway in *S. macrospora* (Engh *et al.* 2007a). Therefore, it seemed likely that the G to A transition in the *tih* gene was the causative mutation in the spore color mutant fus, and that the transition might lead to a splice defect of the second intron.

**Figure 4:**
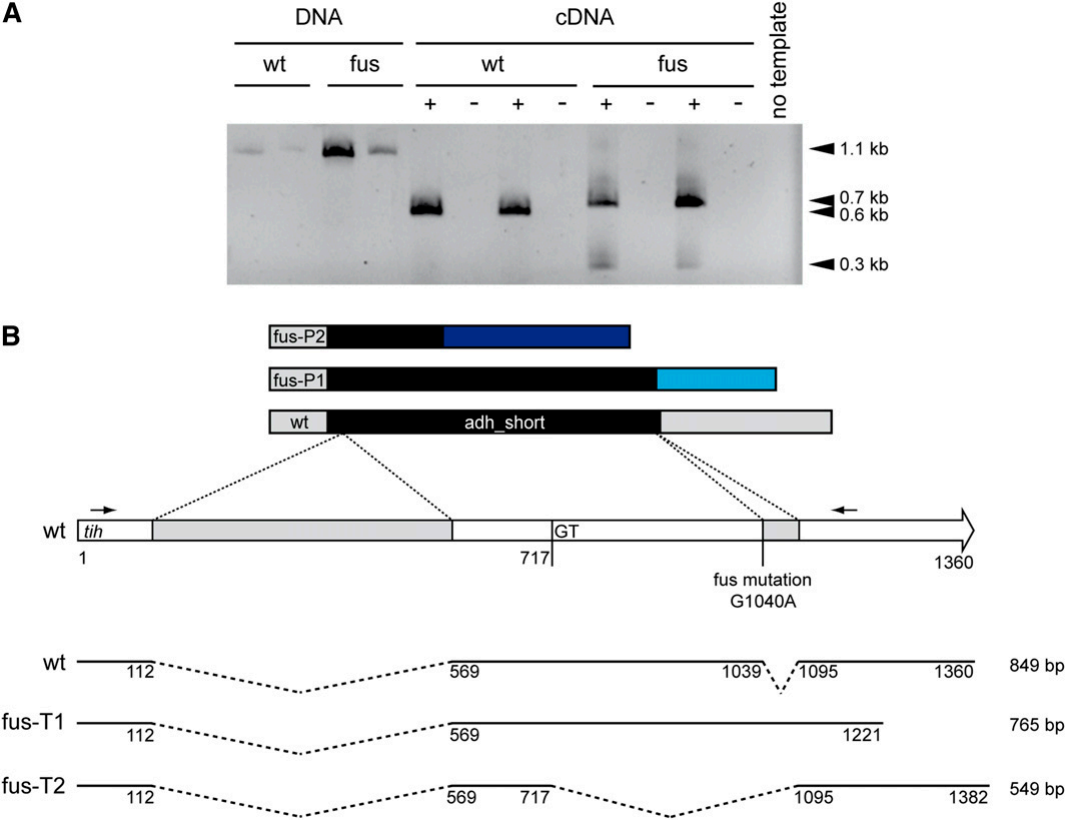
A point mutation in the *tih* gene causes a splice defect in the fus mutant strain. (A) PCR and reverse-transcription PCR analysis of the *tih* gene in the wild type and fus mutant. Small arrows in (B) indicate the oligonucleotide primers used to amplify a *tih* gene fragment. “+” and “−” indicate experiments with or without reverse transcriptase, respectively. Each sample was analyzed in duplicate with two independent biological replications. As expected, a 1.1-kb fragment was amplified from genomic DNA of both strains. From wild-type cDNA, the expected 0.6-kb fragment (after splicing of two introns) was amplified. PCR from cDNA of the fus strain yielded two fragments of 0.7 and 0.3 kb. (B) Two misspliced *tih* transcripts were identified in the fus mutant strain. The wild-type *tih* gene is shown as a white arrow with introns indicated in gray. Below are the derived coding regions, and above predicted peptides from the wild type and mutant fus. Sequence analysis of the PCR fragments from (A) confirmed the point mutation in the first base of the second intron in the fus mutant (nt 1040, G to A transition). The mutation leads to a splice defect of the second intron resulting in two transcripts in the fus strain (fus-T1 and fus-T2). In the first transcript (fus-T1), the second intron is not spliced, leading to a longer transcript represented by the 0.7 kb RT-PCR fragment in (A), and a premature stop codon, and therefore a shorter coding sequence, and a frame-shifted, shorter peptide (indicated in light blue in peptide fus-P1). In the fus-T2 transcript, a second intron is spliced using an incorrect 5′ splice site at nt 718, which results in a shorter transcript represented by the 0.3 kb RT-PCR fragment in (A), and a shorter, frame-shifted peptide (indicated in dark blue in peptide fus-P2). The lengths of the coding sequences of the wild-type and the two fus transcripts are given on the right; adh_short indicates the alcohol dehydrogenase domain within the predicted TIH peptide which is not fully present in the derived peptides fus-P1 and fus-P2 of the mutant strain.

**Figure 5:**
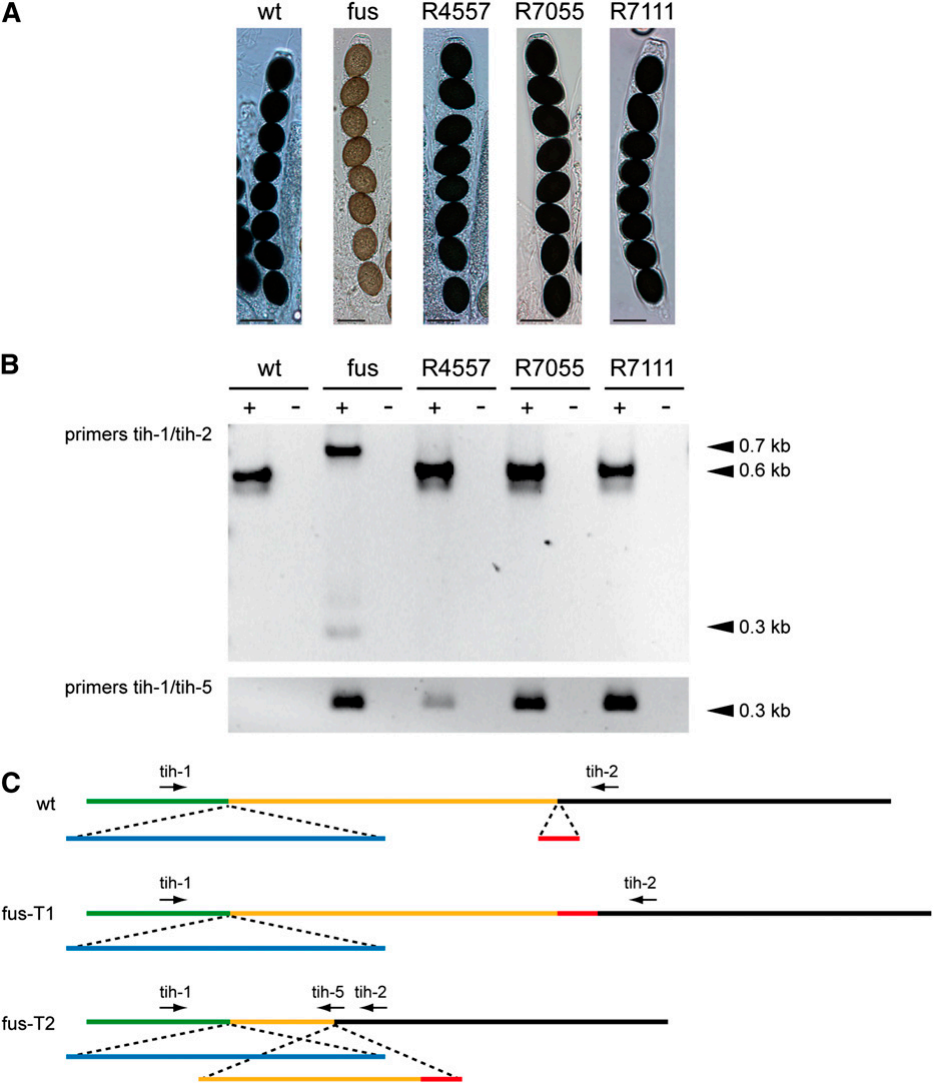
Complementation of the fus mutant with a wild-type copy of the *tih* gene. Mutant fus was transformed with plasmid pEHN5-tih that carries the *tih* ORF under control of respecitve *gpd* and *trpC* promoter and terminator sequences of *A. nidulans*. Single spore isolates from three independent complemented transformants (R4557, R7055, and R7111) were analyzed for phenotype and expression of the *tih* gene. (A) Strains were grown on corn meal agar, and photographs of ascospores were taken after 9 d. The fus mutant has light-brown ascospores whereas the wild type and the complemented transformants have black ascospores. Scale bars indicate 20 μm. (B) RT-PCR analysis of *tih* expression. The three complemented strains express the spliced wild-type transcript of the *tih* gene (0.6-kb fragment with primers tih-1/2, position of primers indicated in panel C), whereas in the fus recipient strain, only the misspliced/non-spliced (0.3 kb and 0.7 kb, respectively) fragments were detected. To demonstrate that the complemented transformants still produce the misspliced transcripts (in addition to the complementing wild-type copy), RT-PCR analysis was performed with oligonucleotides tih-1/5 that specifically amplify the misspliced transcript. The corresponding fragment (0.3 kb) can be amplified from the fus strain and the transformants, but not from the wild type. (C) Position of the oligonucleotide primers and the spliced introns in the wild type transcript, and the two transcripts from the mutant strain.

To confirm this hypothesis, we first sequenced a PCR amplicon of part of the *tih* gene from genomic DNA of the fus strain. As expected, the first base of the second exon exhibited a G to A transition in the mutant strain (data not shown). We subsequently tested whether this mutation leads to a splice defect (Figure 4). RT-PCR and sequencing of the PCR fragments showed two introns in the *tih* gene; the first is spliced correctly in both the wild type and the fus mutant strain, whereas the second intron is either not spliced at all in the mutant, or an incorrect 5′ splice site is used leading to a shorter transcript. In both cases, the predicted TIH polypeptide is shorter and carries incorrect C-terminal sequences as the result of frame shifts in the non-spliced or misspliced transcripts. This makes it probable that the fus mutant does not generate functional TIH and therefore cannot produce melanin. To further verify that the mutation in the *tih* gene is the causative mutation, we transformed the fus mutant with a construct expressing the wild type *tih* gene. The resulting transformants carrying the wild-type *tih* exhibited the wild-type phenotype and produced correctly spliced transcript, in addition to the misspliced versions (Figure 5). These results demonstrate that indeed the fus mutant phenotype is caused by a mutation in the second intron of *tih*, resulting in a splice defect in the mutant strain.

### Analysis of the causative mutation in mutant pro44

**Figure 6:**
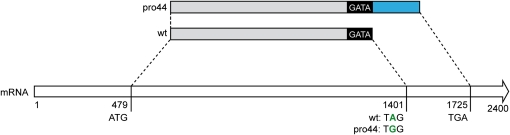
A point mutation in the *pro44* gene (*SMAC_03223*) shifts the stop codon in the mutant strain. Schematic representation of the 2.4-kb *pro44* mRNA and the derived wild-type and mutant proteins (above). The A to G mutation in the wild-type stop codon leads to the formation of a longer polypeptide in the mutant strain. The additional 107 amino acids are shown in blue; the predicted GATA zinc finger domain is shown in black.

**Figure 7:**
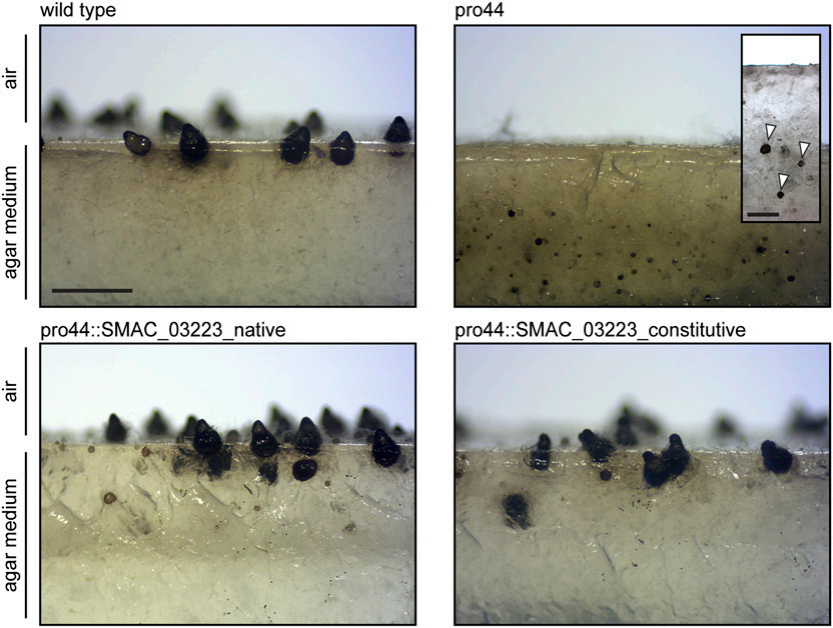
Complementation of mutant pro44 with constructs containing *SMAC_03223*. Mutant pro44 was transformed with plasmid pIG3146-37 that carries the *SMAC_03223* ORF under control of its own promoter and terminator sequences (transformant pro44::SMAC_03223_native) or plasmid pIG3147-1 that carries the *SMAC_03223* ORF under control of the respective *gpd* and *trpC* promoter and terminator sequences of *Aspergillus nidulans* (transformant pro44::SMAC_03223_constitutive). The figure shows a side view (longitudinal section) of the region comprising the agar/air interface from cultures of the wild type, the sterile mutant pro44 and two complemented transformants. The wild type forms mature perithecia at the agar/air interface, whereas mutant pro44 only forms protoperithecia that are submerged in the agar (greater magnification shown in small inserted photograph, arrowheads indicate protoperithecia). Complemented transformants produce mature perithecia at the agar/air interface like the wild type; however, some protoperithecia and even mature perithecia are still formed submerged in the growth medium. Strains were grown on corn meal agar; photographs were taken after 8 d; scale bar indicates 1 mm, and 200 µm in the inserted pro44 photograph.

Bioinformatics analysis of small variants yielded seven point mutations present in pro44 but not in the other samples. Among the mutations, only one changed a coding region: A mutation within the stop codon (A to G) of *SMAC_03223* turns the stop codon into an amino acid-encoding codon resulting in a 107 amino acid extension of the predicted protein (Figure 6). To demonstrate that this mutation is the causative mutation leading to sterility in mutant pro44, the mutant was transformed with plasmids encoding the *SMAC_03223* ORF under control of its own regulatory regions, or under control of the *A. nidulans gpd* promoter and *trpC* terminator. All constructs were able to complement the mutant to fertility, confirming that the sterile phenotype of mutant pro44 is attributable to the defective *SMAC_03223* gene (Figure 7). Therefore, *SMAC_03223* was assigned the name *pro44*.

Interestingly, the complemented transformants, although again producing fully fertile fruiting bodies, retained some mutant characteristics, namely the production of sexual structures within the agar medium. Whereas the wild type forms protoperithecia and later mature perithecia at the agar/air interface, mutant pro44 only forms protoperithecia, and these are completely embedded in the medium (Figure 7). The complemented transformants form mature perithecia not only at the agar/air interface as in the wild type but also within the medium. Thus, the fruiting body maturation process was fully restored in the complemented transformants, whereas the correct distribution of sexual structures was not restored in all cases. However, it must be considered that the transformants still produce the mutated version of the protein that could exert aberrant functions, in this case resulting in mislocalization of fruiting bodies.

## Discussion

### Whole-genome sequencing of mutant strains is a rapid method to identify causative mutations even without prior mapping information

Advances in sequencing technologies and bioinformatics now make it possible to sequence even eukaryotic genomes quickly and at relatively moderate costs. Therefore, many genomes from a single species can be sequenced, and recent reports for several species demonstrate that mutations can be identified that cause specific phenotypes or diseases (Irvine *et al.* 2009; Lupski *et al.* 2010; Pomraning *et al.* 2011; Roach *et al.* 2010; Sarin *et al.* 2008; Schneeberger *et al.* 2009). However, the strategies applied in these studies often included previous mapping of the mutation to a smaller genomic region and/or resequencing of large numbers of candidate mutations to distinguish between true mutations and sequence errors. Recently, two studies were performed in *S. pombe* and *C. elegans* to identify mutations through whole-genome sequencing without previous mapping and without crossing of strains with different genetic backgrounds (Irvine *et al.* 2009; Zuryn *et al.* 2010). Similar to our work, in the *C. elegans* study mutants were backcrossed against the wild type, and more than one mutant genome was sequenced simultaneously. This strategy allows for easier elimination of sequence deviations caused by sequencing errors in the sequenced or the reference genomes. In our study, the robustness of the assay was increased further by using pooled DNA from 40 single spore isolates with the same phenotype instead of sequencing individual genomes of single progeny. In contrast to the analysis in *C. elegans* (Zuryn *et al.* 2010), we could not use an accumulation of mutagen-induced variants to indicate the region carrying the mutation in the progeny of backcrossed mutants, because the mutant strains used here had already been backcrossed several times to reduce the mutational load induced during mutagenesis. However, this fact and the use of pooled DNA from progeny with the same phenotype ensured that overall, the number of true sequence differences in comparison with the reference sequence was small. The true differences mostly consisted of sequencing errors in the reference genome that were easily identified because they occurred in all sequenced samples, including the resequenced wild type.

Prior to our study, only two mutant sequencing approaches to identify mutations responsible for a specific phenotype were reported for filamentous fungi, both of them for *N. crassa* (McCluskey *et al.* 2011; Pomraning *et al.* 2011). Pomraning *et al.* (2011) crossed a mutant with a wild-type strain with different genetic background; McCluskey *et al.* (2011) used previous mapping information to narrow the genomic regions to be screened for mutations. However, such resources are not available for the majority of filamentous fungi, including *S. macrospora*. In the analysis described here, we used a different approach to show that identification of mutations through whole-genome sequencing is possible, even in the absence of prior mapping information, or strains with different genetic backgrounds.

### Sequenced mutant genomes carry mutations in three developmental genes

Although the mutants that were used in this study were all generated by EMS mutagenesis, the causative mutation in pro23 is quite different at the molecular level from those in pro44 and fus. The mutations in mutant fus and pro44 are transitions, which are typical for EMS-induced changes (Bautz and Freese 1960). In contrast, the mutation in pro23 consists of a combination of deletion, duplication, and inversion (Figure 2) at the *pro41* locus. This type of mutation is not typical for EMS mutagenesis; however, our previous studies of mutant pro41 (also a product of EMS mutagenesis) showed that this mutant carries a 4-kb deletion that includes the *pro41* ORF (Nowrousian *et al.* 2007). This finding indicates that the *pro41* gene locus might be prone to this type of mutation. Another possibility is that the rearrangements detected in mutant pro23 arose from a replication-based mechanism recently described to explain complex structural variants in the human genome (Conrad *et al.* 2010; Lee *et al.* 2007; Zhang *et al.* 2009). It was proposed that when the replication fork stalls, *e.g.* due to single-strand breaks or single nucleotide deletions, repeated template switching to nearby genomic regions can occur, leading to apparent deletions when parts of the template are skipped, and to duplications when the replication machinery “jumps back”. One consequence of the fact that the mutations found after EMS mutagenesis are apparently not restricted to point mutations is that both the sequencing strategy, and the subsequent bioinformatics analyses should be optimized not only to find small variants but also larger structural variations in the range of several kilobases.

The rearrangements in mutant pro23 within the genomic region comprising the *pro41* gene lead to a defective *pro41* as demonstrated by the fact that the wild-type copy of *pro41* is able to rescue the pro23 mutant phenotype. The developmental gene *pro41* was first identified through the analysis of the pro41 mutant (Nowrousian *et al.* 2007). The identification of an allelic *pro41* mutation as the causative mutation in the independent mutant pro23, which originated from a different round of EMS mutagenesis, indicates that our mutant collection of ∼100 sterile mutants might reach saturation (Kück *et al.* 2009).

The identification of a point mutation leading to a splice defect in the *tih* gene is the first case of a mutation within an intron sequence that results in a mutant phenotype in *S. macrospora*. The mutants characterized previously either carry deletions that cover complete ORFs (Masloff *et al.* 1999; Nowrousian *et al.* 2007), or point mutations within exons that lead to amino acid exchanges or premature stop codons (Bloemendal *et al.* 2010; Engh *et al.* 2007b; Nowrousian *et al.* 1999; Pöggeler and Kück 2004). A similar mutation of G to A at the 5′ end of an intron was described in the developmental mutant grisea of *Podospora anserina* (Borghouts *et al.* 1997).

The mutation in the *tih* gene leads to a shorter/altered C-terminus in the derived TIH protein that is apparently not functional in the fus mutant, evidenced by brownish pigments instead of black melanins. TIH is predicted to catalyze the fourth enzymatic step in the biosynthesis of DHN (dihydroxynaphtalene) melanins (Bell and Wheeler 1986). *tih* and three other genes (*pks*, *sdh*, and *teh*) involved in melanin biosynthesis are transcriptionally up-regulated during sexual development, and down-regulated in mutants blocked early during this process (Engh *et al.* 2007a; Nowrousian *et al.* 2005). *pks* and *sdh* were already shown to be essential for melanin biosynthesis, and this study demonstrates that *tih* is also required for the melanin formation that gives rise to the black color of the *S. macrospora* ascospores.

In mutant pro44, a mutation within the stop codon of the *pro44* gene leads to a predicted protein with an additional 107 amino acids at the C-terminus. The wild type C-terminus of PRO44 is the most conserved part of the protein and comprises the predicted DNA binding domain of GATA-type transcription factors. Therefore, it seems likely that the additional amino acids derived from a usually noncoding genomic region cause structural changes that prevent function of the mutant protein. *pro44* orthologs have been characterized in *A. nidulans*, *N. crassa*, and *A. fumigatus*, where they were shown to be essential for fruiting body formation (Colot *et al.* 2006; Han *et al.* 2001; Szewczyk and Krappmann 2010). Furthermore, the *N. crassa* deletion strain of the *pro44* ortholog *sub-1* produces protoperithecia that are submerged in the agar medium as opposed to the production of protoperithecia at the agar/air interface in the wild type (Colot *et al.* 2006). This phenotype is similar to what we observed in pro44 (Figure 7). Thus, *pro44* encodes a conserved transcription factor essential for sexual development in filamentous ascomycetes. *pro44* is the second transcription factor-encoding gene that was identified from the pro mutant-screen; the first being the zinc cluster-type transcription factor encoded by *pro1* (Masloff *et al.* 1999).

### Conclusions

The results presented here demonstrate that for small eukaryotic genomes, whole-genome sequencing of mutant strains is a fast and efficient means to discover mutations that are responsible for specific phenotypes. The strategy used here requires that the mutants can be genetically crossed against other strains, but it does not require any prior mapping information, or strains with different genetic backgrounds. Therefore, this method will be useful for the growing number of species that are used as experimental systems to study a wide range of biological phenomena, but for which only a small range of genetic and molecular resources is available.

## Supplementary Material

Supporting Information
