# Supplementary material for: Whole-Genome Sequencing of Sordaria macrospora Mutants Identifies Developmental Genes
Source: G3 (Bethesda). 2012 Feb 1;2(2):261–70. doi: 10.1534/g3.111.001479 (PMC3284333; doi:10.1534/g3.111.001479)
Supplement: Supporting Information [file supp_2_2_261__index.html]

Supporting Information 

# Whole-Genome Sequencing of *Sordaria macrospora* Mutants Identifies Developmental Genes

## Supporting Information for Nowrousian *et al.*, 2012

**Files in this Data Supplement:**

- Supporting Information - Figures S1-S3 and File S1 (PDF, 778 KB)
- Figure S1 - Crossing history for the mutants used in this study (PDF, 227 KB)
- Figure S2 - Strategy for whole genome-sequencing of pooled DNA from mutant pro44 (PDF, 205 KB)
- Figure S3 - Coverage and variant frequencies for the sequenced wild type, pro23/fus, and pro44 samples (PDF, 590 KB)
- File S1 - Supporting methods(PDF, 318 KB)
