# Supplementary material for: Whole-Genome Sequencing of Sordaria macrospora Mutants Identifies Developmental Genes
Source: G3 (Bethesda). 2012 Feb 1;2(2):261–70. doi: 10.1534/g3.111.001479 (PMC3284333; doi:10.1534/g3.111.001479)
Supplement: Supporting Information [file supp_2.2.261_001479SI.pdf]

SUPPORTING INFORMATION (Figure S1, Figure S2, Figure S3, File S1)

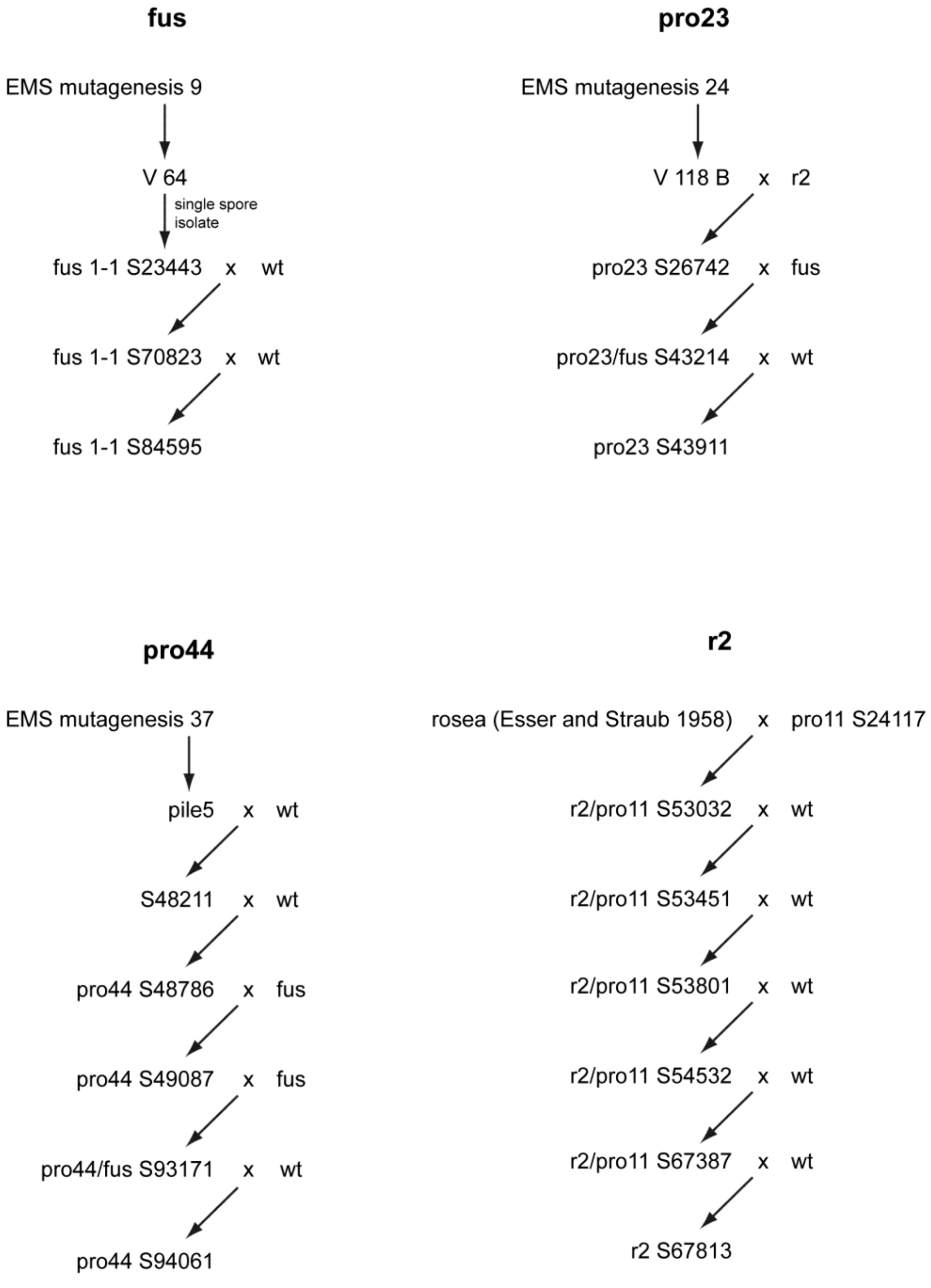

Figure S1 Crossing history for the mutants used in this study. Strains were backcrossed against the wild type (wt) or the spore color mutants fus and r2, both of which are fertile but produce light-brown and red spores, respectively, instead of black spores.

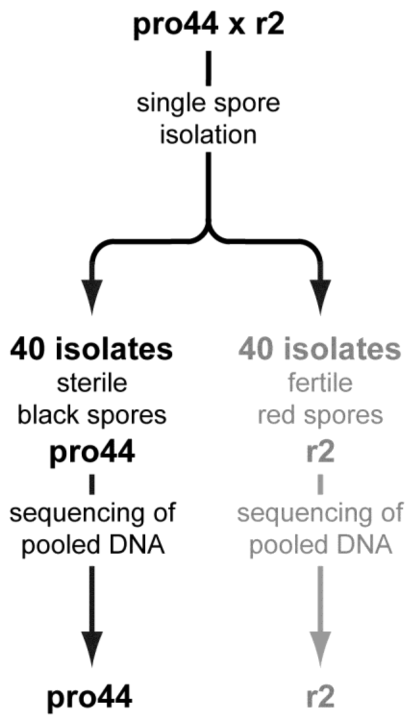

Figure S2 Strategy for whole genome-sequencing of pooled DNA from mutant pro44. Mutant pro44 was crossed against the spore color mutant r2. Single spore isolates arising from both black and brown-red ascospores were screened for fertility and color, and 40 spores with the phenotype sterile/black spores were chosen to represent mutant pro44. The pooled DNA from these 40 spore isolates was used for sequencing.

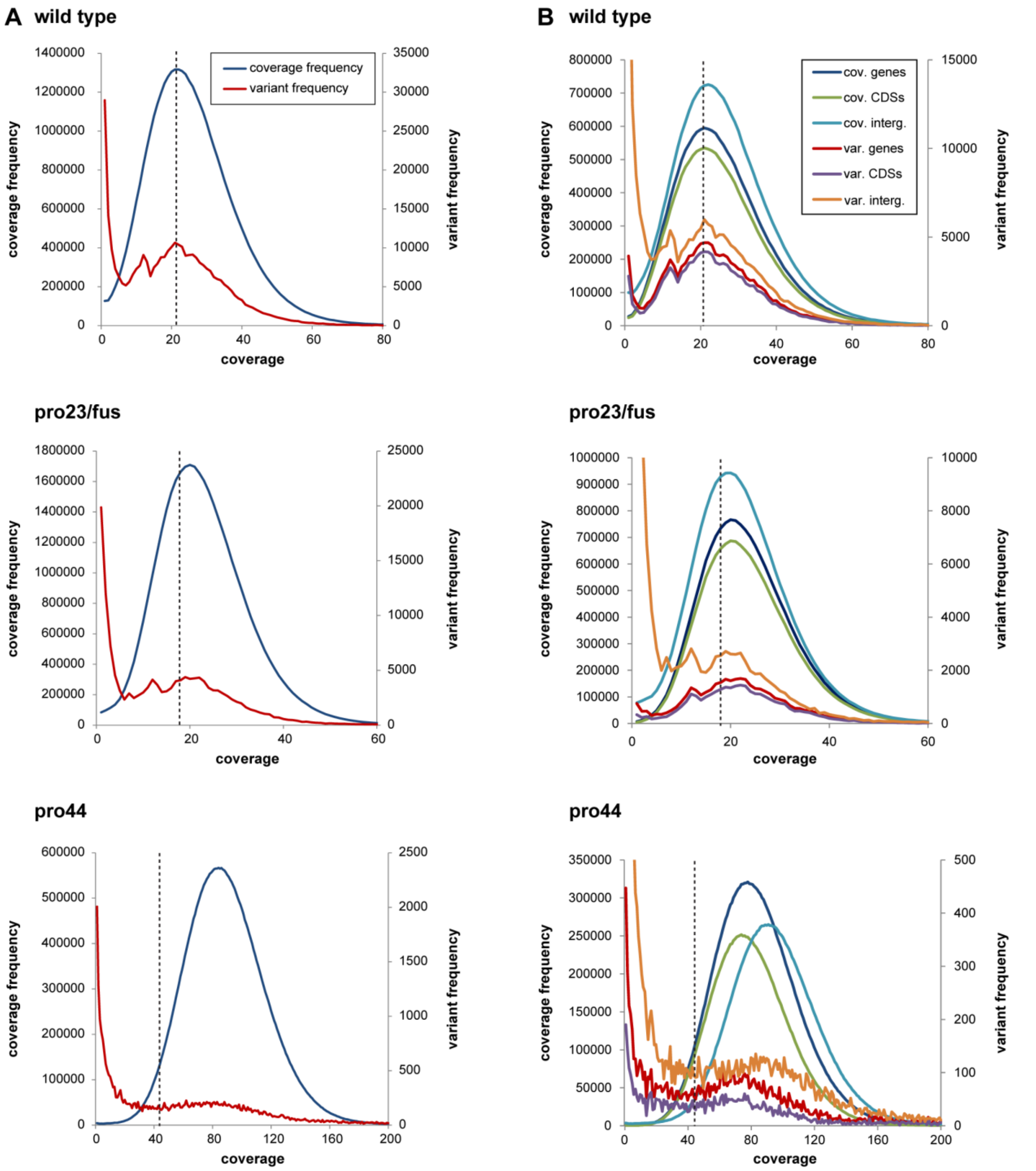

Figure S3 Coverage and variant frequencies for the sequenced wild type, pro23/fus, and pro44 samples. Custom-made Perl scripts were used to determine the read coverage for each base of the genome sequence from the results of the pileup function of SAMtools (Li et al. 2009 Bioinf 25:2078-2079), and to calculate coverage frequencies (y-axis on the left of each graph). In addition, it was determined for each coverage value how many bases with this coverage were identified as variant bases by SAMtools (variant frequency, y-axis on the right of each graph). Bases with a coverage of >1000 were set to 1000, graphs are shown for coverages from 1 to 60, 80, or 200, depending on the average coverage of the sample. Coverages above these values did not contribute significantly to overall coverage. Peak coverages in this analysis are somewhat lower than the average coverages given in Table 2 (main manuscript), because the latter were calculated from the cleaned reads prior to mapping. In **A** (left side), coverage frequencies across the whole genome are shown, in **B** (right

side), coverage frequencies were determined for bases that are annotated as genes (ORFs including introns and UTRs), CDSs, or intergenic regions. The coverage cutoff that was used in our analyes to search for small variants is indicated by a vertical dashed line. The analyses show that the variant frequencies are rather high for bases with low coverage, most likely these bases are in regions that are difficult to sequence due to low sequence complexity, extreme base compositions, secondary structures etc., and represent sequencing errors rather than true sequence variants. This might also be the reason why intergenic regions (which contain more low complexity regions etc. than genes or CDSs) consistently have a higher variant frequency than genes or CDSs, even at coverage values where the intergenic regions have a lower coverage frequency than the other regions. Overall, these data show that a coverage cutoff is needed to avoid calling numerous false-positive variants. For our analysis, we set a relatively high coverage cutoff to avoid false-positives variants to a large degree.

File S1 Supplementary methods

Read mapping and extraction of small variants

Mapping of reads onto reference genome was done with BWA (Li and Durbin 2009 Bioinf 25:1754-1760), and extraction of small sequence variants was done using SAMtools (Li et al. 2009 Bioinf 25:2078-2079). Briefly, SAM files were converted to BAM files, and small sequence variants (single nucleotide polymorphisms, insertions/deletions) were identified using the SAMtools pileup and filtering functions (see below). The reads that were used were cleaned previously to remove all reads with undetermined bases ("N"). Therefore, not all reads from the mate-pair sequencing have a "partner" any more, these reads were collected in a separate file for single reads.

a) mapping of reads onto reference genome with BWA

- create index for the S. macrospora genome with:

```
./bwa index Sm_01_genome.fasta
```

- map reads onto the *S. macrospora* genome (separately for paired-end data and single reads with `aln`, runs are combined only in the `sampe` step):

```
./bwa aln Sm_01_genome.fasta NG-5090_23_1_sequence_trimmed.fastq >NG-
5090_23_1_sequence_trimmed.aln
```

for single reads:

```
./bwa samse Sm_01_genome.fasta NG-5090_23_sequence_singlets.aln NG-
5090_23_sequence_singlets.fastq >NG-5090_23_sequence_singlets.sam
```

for paired-end reads:

```
./bwa sampe Sm_01_genome.fasta NG-5090_23_1_sequence_trimmed.aln NG-
5090_23_2_sequence_trimmed.aln NG-5090_23_1_sequence_trimmed.fastq NG-
5090_23_2_sequence_trimmed.fastq >NG-5090_23_sequence_trimmed.sam
```

b) downstream processing with SAMtools

- create index Sm_01_genome.fasta.fai for reference sequence with:

```
./samtools faidx Sm_01_genome.fasta
```

- import SAM file from BWA and convert to BAM file with:

```
./samtools import Sm_01_genome.fasta.fai NG-5090_23_sequence_trimmed.sam
NG-5090_23_sequence_trimmed.bam
```

- sort BAM file (necessary for fast access, sorted by reference sequence contigs etc.) with:

```
./samtools sort NG-5090_23_sequence_trimmed.bam NG-
5090_23_sequence_trimmed_sorted.bam
```

- merge BAM files for paired-end and singlets with:

```
./samtools merge NG-5090_23_all_sorted.bam NG-
5090_23_sequence_trimmed_sorted.bam NG-
5090_23_sequence_singlets_sorted.bam
```

- create index NG-5090_23_sequence_trimmed_sorted.bam.bai from sorted BAM file with:

```
./samtools index NG-5090_23_all_sorted.bam
```

for graphical view, start text viewer with:

```
./samtools tview NG-5090_23_all_sorted.bam Sm_01_genome.fasta
```

in the viewer mode, get help with "?", go to region (e.g. scaffold_1:4,000) with "g", quit with "q"

- for variant callling (SNPs, indels) use pileup (-c option for consensus sequence, -v option: only variants are reported, not complete consesus for all bases, even those that are the same as the reference):

```
./samtools pileup -f Sm_01_genome.fasta -vc NG-5090_23_all_sorted.bam
>NG-5090_23_all_sorted_pileup.txt
```

- filter raw variant calls with samtools.pl varFilter. Set -D option (maximum read depth) according to the average read depth. SNPs with excessively high read depth are usually caused by structural variations or alignment artifacts and should not be trusted.

```
./samtools.pl varFilter -D100 NG-5090_23_all_sorted_pileup.txt >NG-
5090_23_all_sorted_pileup_filter.txt
```

- Acquire final variants by setting a quality threshold with awk. Here, the quality threshold for indels is 25 and 10 for substitutions.

```
awk '($3=="*"&&$6>=25)||($3!="*"&&$6>=10)' NG-
5090_23_all_sorted_pileup_filter.txt >NG-
5090_23_all_sorted_pileup_filter_threshold.txt
```

- for determining the consensus sequence use pileup with -c option:

```
./samtools pileup -f Sm_01_genome.fasta -c NG-5090_23_all_sorted.bam >NG-
5090_23_all_sorted_pileup_consensus.txt
```

- for retaining only those lines with a consensus base (and not those with indels [consensus = *] that were already found by pileup):

```
awk '($3~/^[acgtnACGTN]$/)' NG-5090_23_all_sorted_pileup_consensus.txt
>NG-5090_23_all_sorted_pileup_consensus_only_bases.txt
```

- Further processing was done with custom-made Perl scripts or manually.

Analysis of large insertions/deletions and inversions using mate-pair information

a) Analysis of large insertions/deletions

One way to check for large putative insertions or deletions is to check for deviations from the expected distance of paired reads. For this purpose, information from the SAM files resulting from mapping with BWA (see Supplementary method S1) were used. The second column in each SAM file contains a flag that gives information about the mapping of a read and its mate (e.g. whether one or both reads are mapped, the strand of read and mate etc.) (Li et al. 2009 Bioinf 25:2078-2079). Flags that indicate correct (i.e. expected) mapping for mate-pair reads (i.e. oriented away from each other on opposite strands) are 81, 83, 145 and 147 if the insert size (given in the 9th column of the SAM file) is positive. These conditions were used to extract reads from the SAM files where both reads of a pair map onto the same contig/scaffold in the correct orientation with:

```
awk
'($2=="145"||$2=="81"||$2=="83"||$2=="147")&&($7=="=")&&($3!="*")&&($9>0)'
NG-5090_23_sequence_trimmed.sam >NG5090_23_sequence_for_indel_size.sam
```

The resulting reduced SAM file was processed further with custom-made Perl scripts to perform a sliding window analysis with a window size of 500 bp where for each window the average insert size for all mate-pairs, standard deviation and coefficient of variance are calculated. Only reads that map perfectly (no mismatches) were used, duplicated reads (with the same mapping position for read and mate) were excluded. The results can be searched for deviations from the average insert size that can indicate deletions (larger insert size in the mapping results) or insertions (smaller insert size in the mapping results).

b) Analysis of inversions within scaffolds/contigs

Similar to the case of insertions/deletions, mate-pair information from SAM files can also be used to find cases of putative inversions within contigs/scaffolds. For this purpose, all mate-pairs were extracted where both reads map in the same direction on a contig/scaffold:

```
awk '(($2=="65"||$2=="113")&&($7=="=")&&($3!="*")&&($9!="0"))' NG-
5090_23_sequence_trimmed.sam
```

Further processing (sliding window analysis of reads with calculation of average, standard deviation and coefficient of variance of the predicted insert size) was done with custom-made Perl scripts.
