# Supplementary material for: Whole-Genome Sequencing of Sordaria macrospora Mutants Identifies Developmental Genes
Source: G3 (Bethesda). 2012 Feb 1;2(2):261–70. doi: 10.1534/g3.111.001479 (PMC3284333; doi:10.1534/g3.111.001479)
Supplement: Supporting Information [file supp_2.2.261_FigureS1.pdf]

SUPPORTING INFORMATION (Figure S1, Figure S2, Figure S3, File S1)

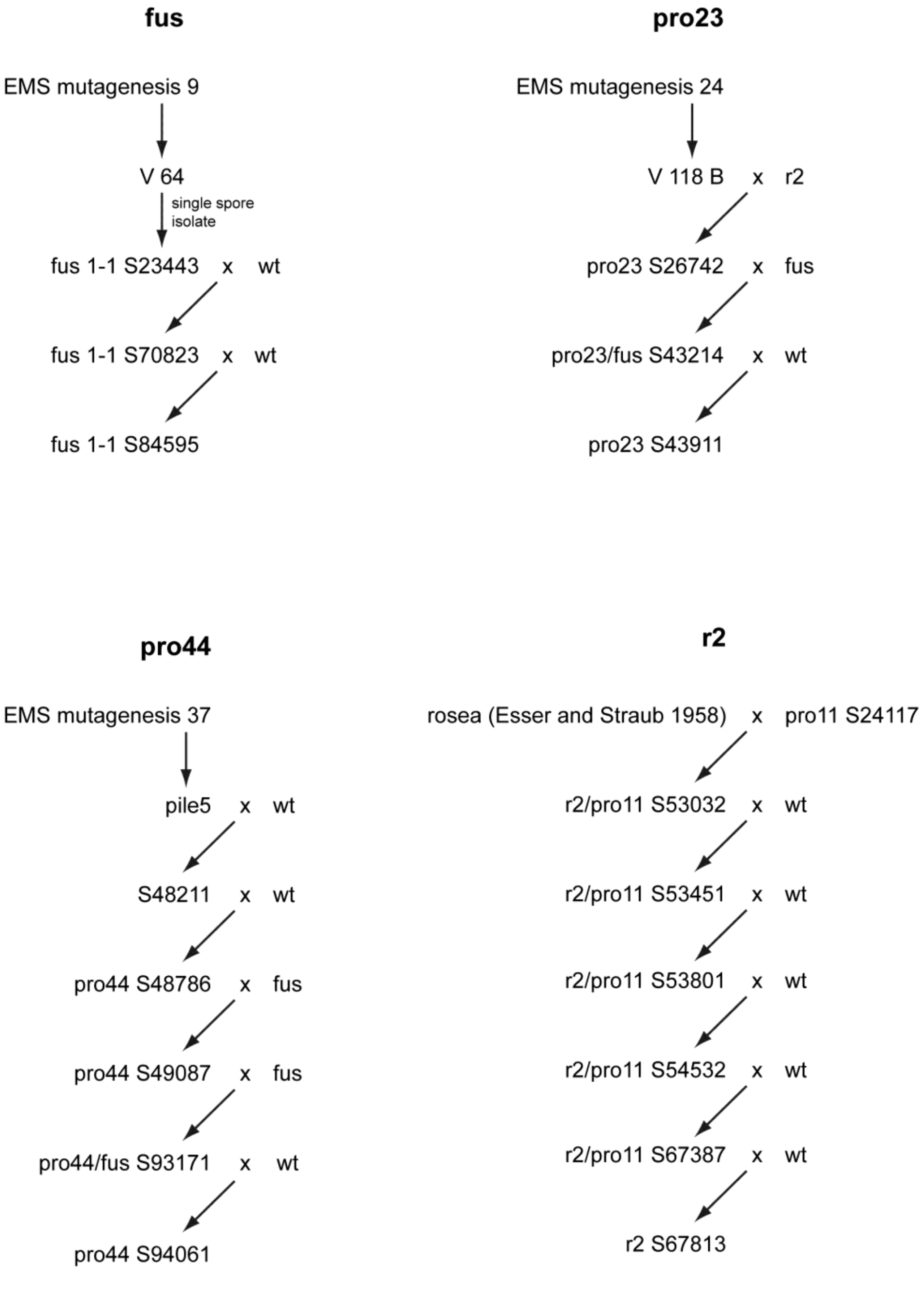

Figure S1 Crossing history for the mutants used in this study. Strains were backcrossed against the wild type (wt) or the spore color mutants fus and r2, both of which are fertile but produce light-brown and red spores, respectively, instead of black spores.
