# Supplementary material for: Whole-Genome Sequencing of Sordaria macrospora Mutants Identifies Developmental Genes
Source: G3 (Bethesda). 2012 Feb 1;2(2):261–70. doi: 10.1534/g3.111.001479 (PMC3284333; doi:10.1534/g3.111.001479)
Supplement: Supporting Information [file supp_2.2.261_FigureS2.pdf]

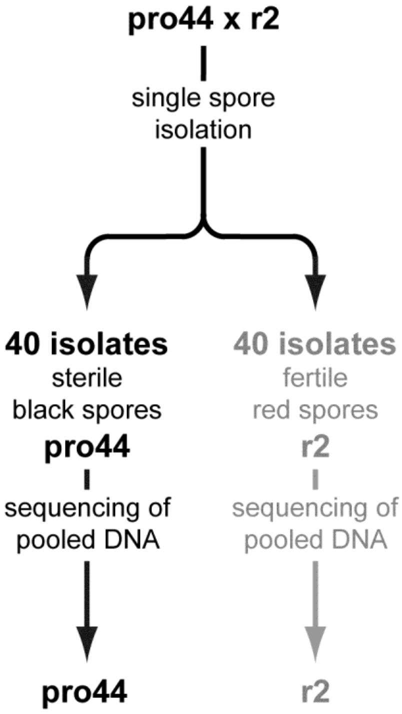

Figure S2 Strategy for whole genome-sequencing of pooled DNA from mutant pro44. Mutant pro44 was crossed against the spore color mutant r2. Single spore isolates arising from both black and brown-red ascospores were screened for fertility and color, and 40 spores with the phenotype sterile/black spores were chosen to represent mutant pro44. The pooled DNA from these 40 spore isolates was used for sequencing.
