# Supplementary material for: Whole-Genome Sequencing of Sordaria macrospora Mutants Identifies Developmental Genes
Source: G3 (Bethesda). 2012 Feb 1;2(2):261–70. doi: 10.1534/g3.111.001479 (PMC3284333; doi:10.1534/g3.111.001479)
Supplement: Supporting Information [file supp_2.2.261_FigureS3.pdf]

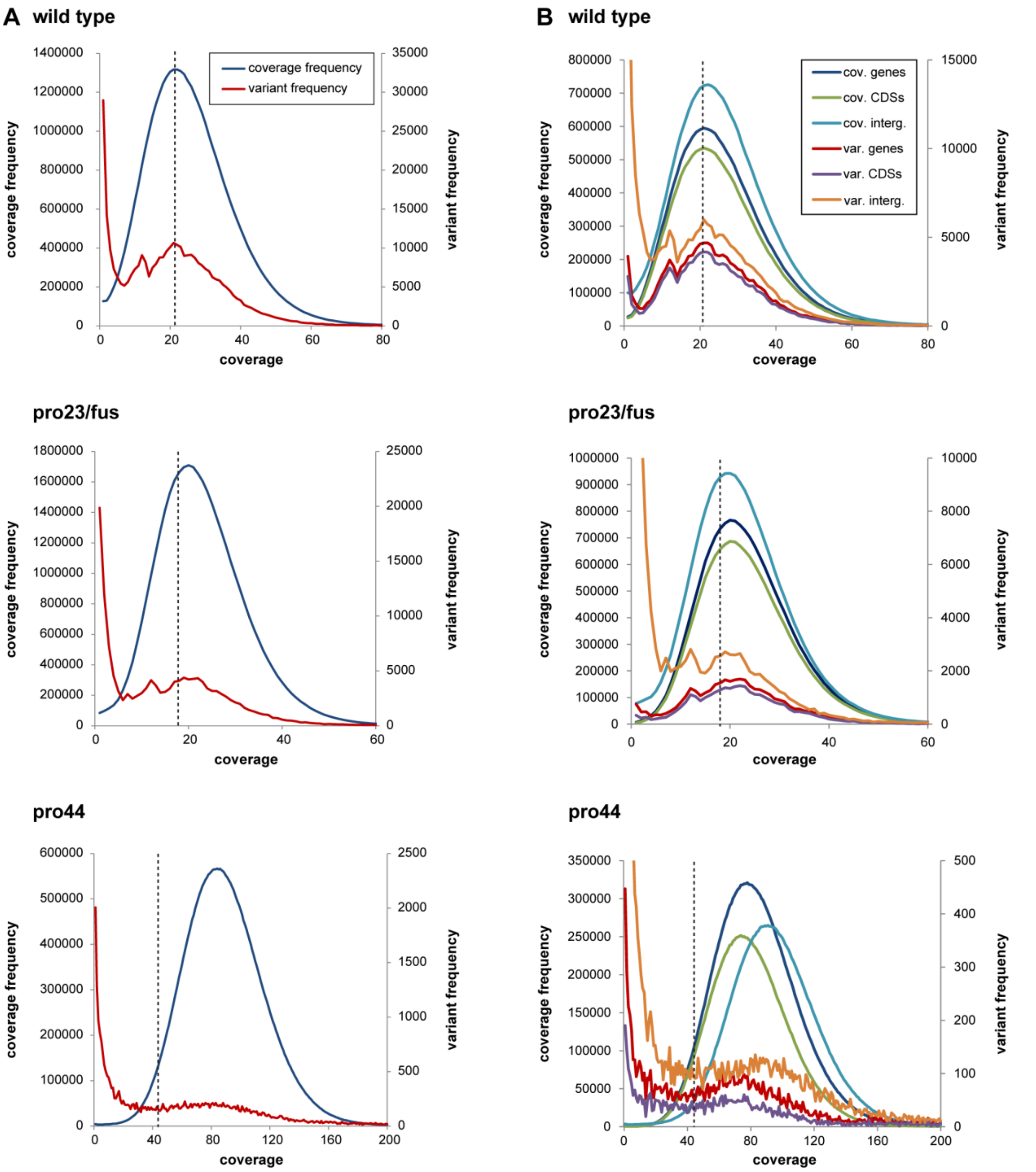

Figure S3 Coverage and variant frequencies for the sequenced wild type, pro23/fus, and pro44 samples. Custom-made Perl scripts were used to determine the read coverage for each base of the genome sequence from the results of the pileup function of SAMtools (Li et al. 2009 Bioinf 25:2078-2079), and to calculate coverage frequencies (y-axis on the left of each graph). In addition, it was determined for each coverage value how many bases with this coverage were identified as variant bases by SAMtools (variant frequency, y-axis on the right of each graph). Bases with a coverage of >1000 were set to 1000, graphs are shown for coverages from 1 to 60, 80, or 200, depending on the average coverage of the sample. Coverages above these values did not contribute significantly to overall coverage. Peak coverages in this analysis are somewhat lower than the average coverages given in Table 2 (main manuscript), because the latter were calculated from the cleaned reads prior to mapping. In **A** (left side), coverage frequencies across the whole genome are shown, in **B** (right

side), coverage frequencies were determined for bases that are annotated as genes (ORFs including introns and UTRs), CDSs, or intergenic regions. The coverage cutoff that was used in our analyes to search for small variants is indicated by a vertical dashed line. The analyses show that the variant frequencies are rather high for bases with low coverage, most likely these bases are in regions that are difficult to sequence due to low sequence complexity, extreme base compositions, secondary structures etc., and represent sequencing errors rather than true sequence variants. This might also be the reason why intergenic regions (which contain more low complexity regions etc. than genes or CDSs) consistently have a higher variant frequency than genes or CDSs, even at coverage values where the intergenic regions have a lower coverage frequency than the other regions. Overall, these data show that a coverage cutoff is needed to avoid calling numerous false-positive variants. For our analysis, we set a relatively high coverage cutoff to avoid false-positives variants to a large degree.
