# Supplementary material for: Whole-Genome Sequencing of Sordaria macrospora Mutants Identifies Developmental Genes
Source: G3 (Bethesda). 2012 Feb 1;2(2):261–70. doi: 10.1534/g3.111.001479 (PMC3284333; doi:10.1534/g3.111.001479)
Supplement: Supporting Information [file supp_2.2.261_FileS1.pdf]

**File S1** Supplementary methods

## Read mapping and extraction of small variants

Mapping of reads onto reference genome was done with BWA (Li and Durbin 2009 Bioinf 25:1754-1760), and extraction of small sequence variants was done using SAMtools (Li et al. 2009 Bioinf 25:2078-2079). Briefly, SAM files were converted to BAM files, and small sequence variants (single nucleotide polymorphisms, insertions/deletions) were identified using the SAMtools pileup and filtering functions (see below). The reads that were used were cleaned previously to remove all reads with undetermined bases ("N"). Therefore, not all reads from the mate-pair sequencing have a "partner" any more, these reads were collected in a separate file for single reads.

### a) mapping of reads onto reference genome with BWA

- create index for the S. macrospora genome with:

```
./bwa index Sm_01_genome.fasta
```

- map reads onto the *S. macrospora* genome (separately for paired-end data and single reads with `aln`, runs are combined only in the `sampe` step):

```
./bwa aln Sm_01_genome.fasta NG-5090_23_1_sequence_trimmed.fastq >NG-
5090_23_1_sequence_trimmed.aln
```

for single reads:

```
./bwa samse Sm_01_genome.fasta NG-5090_23_sequence_singlets.aln NG-
5090_23_sequence_singlets.fastq >NG-5090_23_sequence_singlets.sam
```

for paired-end reads:

```
./bwa sampe Sm_01_genome.fasta NG-5090_23_1_sequence_trimmed.aln NG-
5090_23_2_sequence_trimmed.aln NG-5090_23_1_sequence_trimmed.fastq NG-
5090_23_2_sequence_trimmed.fastq >NG-5090_23_sequence_trimmed.sam
```

### b) downstream processing with SAMtools

- create index Sm_01_genome.fasta.fai for reference sequence with:

```
./samtools faidx Sm_01_genome.fasta
```

- import SAM file from BWA and convert to BAM file with:

```
./samtools import Sm_01_genome.fasta.fai NG-5090_23_sequence_trimmed.sam
NG-5090_23_sequence_trimmed.bam
```

- sort BAM file (necessary for fast access, sorted by reference sequence contigs etc.) with:

```
./samtools sort NG-5090_23_sequence_trimmed.bam NG-
5090_23_sequence_trimmed_sorted.bam
```

- merge BAM files for paired-end and singlets with:

```
./samtools merge NG-5090_23_all_sorted.bam NG-
5090_23_sequence_trimmed_sorted.bam NG-
5090_23_sequence_singlets_sorted.bam
```

- create index NG-5090_23_sequence_trimmed_sorted.bam.bai from sorted BAM file with:

```
./samtools index NG-5090_23_all_sorted.bam
```

for graphical view, start text viewer with:

```
./samtools tview NG-5090_23_all_sorted.bam Sm_01_genome.fasta
```

in the viewer mode, get help with "?", go to region (e.g. scaffold_1:4,000) with "g", quit with "q"

- for variant callling (SNPs, indels) use pileup (-c option for consensus sequence, -v option: only variants are reported, not complete consesus for all bases, even those that are the same as the reference):

```
./samtools pileup -f Sm_01_genome.fasta -vc NG-5090_23_all_sorted.bam
>NG-5090_23_all_sorted_pileup.txt
```

- filter raw variant calls with samtools.pl varFilter. Set -D option (maximum read depth) according to the average read depth. SNPs with excessively high read depth are usually caused by structural variations or alignment artifacts and should not be trusted.

```
./samtools.pl varFilter -D100 NG-5090_23_all_sorted_pileup.txt >NG-
5090_23_all_sorted_pileup_filter.txt
```

- Acquire final variants by setting a quality threshold with awk. Here, the quality threshold for indels is 25 and 10 for substitutions.

```
awk '($3=="*"&&$6>=25)||($3!="*"&&$6>=10)' NG-
5090_23_all_sorted_pileup_filter.txt >NG-
5090_23_all_sorted_pileup_filter_threshold.txt
```

- for determining the consensus sequence use pileup with -c option:

```
./samtools pileup -f Sm_01_genome.fasta -c NG-5090_23_all_sorted.bam >NG-
5090_23_all_sorted_pileup_consensus.txt
```

- for retaining only those lines with a consensus base (and not those with indels [consensus = *] that were already found by pileup):

```
awk '($3~/^[acgtnACGTN]$/)' NG-5090_23_all_sorted_pileup_consensus.txt
>NG-5090_23_all_sorted_pileup_consensus_only_bases.txt
```

- Further processing was done with custom-made Perl scripts or manually.

**Analysis of large insertions/deletions and inversions using mate-pair information**

**a) Analysis of large insertions/deletions**

One way to check for large putative insertions or deletions is to check for deviations from the expected distance of paired reads. For this purpose, information from the SAM files resulting from mapping with BWA (see Supplementary method S1) were used. The second column in each SAM file contains a flag that gives information about the mapping of a read and its mate (e.g. whether one or both reads are mapped, the strand of read and mate etc.) (Li et al. 2009 Bioinf 25:2078-2079). Flags that indicate correct (i.e. expected) mapping for mate-pair reads (i.e. oriented away from each other on opposite strands) are 81, 83, 145 and 147 if the insert size (given in the 9th column of the SAM file) is positive. These conditions were used to extract reads from the SAM files where both reads of a pair map onto the same contig/scaffold in the correct orientation with:

```
awk
'($2=="145"||$2=="81"||$2=="83"||$2=="147")&&($7=="=")&&($3!="*")&&($9>0)'
NG-5090_23_sequence_trimmed.sam >NG5090_23_sequence_for_indel_size.sam
```

The resulting reduced SAM file was processed further with custom-made Perl scripts to perform a sliding window analysis with a window size of 500 bp where for each window the average insert size for all mate-pairs, standard deviation and coefficient of variance are calculated. Only reads that map perfectly (no mismatches) were used, duplicated reads (with the same mapping position for read and mate) were excluded. The results can be searched for deviations from the average insert size that can indicate deletions (larger insert size in the mapping results) or insertions (smaller insert size in the mapping results).

**b) Analysis of inversions within scaffolds/contigs**

Similar to the case of insertions/deletions, mate-pair information from SAM files can also be used to find cases of putative inversions within contigs/scaffolds. For this purpose, all mate-pairs were extracted where both reads map in the same direction on a contig/scaffold:

```
awk '(($2=="65"||$2=="113")&&($7=="=")&&($3!="*")&&($9!="0"))' NG-
5090_23_sequence_trimmed.sam
```

Further processing (sliding window analysis of reads with calculation of average, standard deviation and coefficient of variance of the predicted insert size) was done with custom-made Perl scripts.
